# Prevalence of *Plasmodium *spp. in malaria asymptomatic African migrants assessed by nucleic acid sequence based amplification

**DOI:** 10.1186/1475-2875-8-12

**Published:** 2009-01-12

**Authors:** Marianna Marangi, Rocco Di Tullio, Pètra F Mens, Domenico Martinelli, Vincenzina Fazio, Gioacchino Angarano, Henk DFH Schallig, Annunziata Giangaspero, Gaetano Scotto

**Affiliations:** 1 Dipartimento PrIME, Università di Foggia, Via Napoli 25, 71100 Foggia, Italy; 2 Clinica delle Malattie Infettive, Ospedali Riuniti, Viale Luigi Pinto, 71100 Foggia, Italy; 3 Royal Tropical Institute, Meibergdreef 39, 1105 AZ Amsterdam, The Netherlands; 4 Dipartimento di Scienze Mediche e del Lavoro, Università di Foggia, Viale Luigi Pinto, 71100 Foggia, Italy; 5 Laboratorio di Clinica Chimica, Ospedali Riuniti, Viale Luigi Pinto, 71100 Foggia, Italy

## Abstract

**Background:**

Malaria is one of the most important infectious diseases in the world. Although most cases are found distributed in the tropical regions of Africa, Asia, Central and South Americas, there is in Europe a significant increase in the number of imported cases in non-endemic countries, in particular due to the higher mobility in today's society.

**Methods:**

The prevalence of a possible asymptomatic infection with *Plasmodium *species was assessed using Nucleic Acid Sequence Based Amplification (NASBA) assays on clinical samples collected from 195 study cases with no clinical signs related to malaria and coming from sub-Saharan African regions to Southern Italy. In addition, base-line demographic, clinical and socio-economic information was collected from study participants who also underwent a full clinical examination.

**Results:**

Sixty-two study subjects (31.8%) were found positive for *Plasmodium *using a pan *Plasmodium *specific NASBA which can detect all four *Plasmodium *species causing human disease, based on the small subunit 18S rRNA gene (18S NASBA). Twenty-four samples (38%) of the 62 18S NASBA positive study cases were found positive with a Pfs25 mRNA NASBA, which is specific for the detection of gametocytes of *Plasmodium falciparum*. A statistically significant association was observed between 18S NASBA positivity and splenomegaly, hepatomegaly and leukopaenia and country of origin.

**Conclusion:**

This study showed that a substantial proportion of people originating from malaria endemic countries harbor malaria parasites in their blood. If transmission conditions are available, they could potentially be a reservoir. Thefore, health authorities should pay special attention to the health of this potential risk group and aim to improve their health conditions.

## Background

Malaria is one of the most important infectious diseases in the world. The World Health Organization estimates that 300–500 million cases of malaria infections, with 1–3 million deaths globally occurring each year, of which 95% is caused by *Plasmodium falciparum *[1]. Although most cases are found in the tropical regions of Africa, Asia, Central, and South America, there is a significant increase in the number of imported cases in Europe, in particular due to the higher mobility of people in today's society [2]. Approximately 7,000 cases of imported malaria are recorded in Europe annually [3]. Due to the misleading symptoms related to initial malaria (i.e. fever and other flu-like symptoms), prompt diagnosis and treatment are of great importance. The Centers for Disease Control and Prevention recommends that malaria should be considered in the differential diagnosis of febrile patients who have traveled to a region where malaria is endemic and in any patients who experience fevers of unknown origin regardless of their travel history [2].

The current standard for diagnosis is the microscopic examination of Giemsa-stained thick and thin blood smears [4-8]. This procedure is not expensive and in principle easy to perform, but becomes time-consuming in particular in cases of mixed infection or low parasitaemia and even with experienced microscopists examining the blood slides, misdiagnosis may occur [4,8]. To aid malaria diagnosis, other techniques have been developed, such as immunochromatographic assays based on *Plasmodium *antigen detection, but some of these tests perform poorly in cases of low parasitemia [7,9,10] or, in contrast, lead to a false positive diagnosis of malaria due to the persistence of parasite antigen after adequate treatment [7,11]. Molecular detection for *Plasmodium *with PCR has resulted in increased sensitivity and species discrimination compared to either microscopic or immunochromatographic diagnosis of malaria [12-18]. PCR based assays are sensitive and can be converted to a quantitative format, if SYBR green or molecular probes (e.g. a Taqman probe or a molecular beacon) are used in real time format [19,20]. Alternatively, Nucleic Acid Sequence Based Amplification (NASBA) technology can be applied, which has as advantage above real-time PCR assays that it is fast to perform; i.e. 60 minutes for NASBA compared to up to four hours for real-time PCR [21,22]. NASBA assays, using molecular beacons as detection probes, has been developed for different *Plasmodium *species and has shown to be very sensitive with a detection limit of 20 parasites/ml blood [22,23].

In the present study, NASBA using primers based on pan species *Plasmodium*-specific small sub-unit ribosomal RNA gene sequences [24] was used to evaluate the prevalence of *Plasmodium *species in a population of migrants. Furthermore, a gametocyte specific NASBA based on the detection of the mRNA encoding for the *P. falciparum *gametocyte specific Pfs25 gene [25] was employed to detect mature sexual transmission stages in the same study population.

## Methods

### Patient enrolment, information and sample collection, microscopy

One hundred and ninety five blood samples were collected during the period April-September 2007 from study cases who were temporarily guests in a refugee camp managed by the Italian Red Cross, located in Borgo Mezzanone (province of Foggia, Italy). All guests were orally informed about the purpose of the study and invited to participate. Subsequent recruitment was on voluntary basis with no special inclusion criteria.

The study was reviewed and approved by the local Chief of the Red Cross and written informed consent was obtained from each study subject who was enrolled in the study. All study procedures were in agreement with the Helsinki Declaration (Edinburgh 2000). At enrolment, all study participants were interviewed using a questionnaire to obtain baseline demographic, clinical, and socioeconomic information and to assess their previous exposure to malaria. All enrolled subjects also received a full clinical examination and were treated accordingly.

### Anamnestic data

The mean age of the enrolled study subjects (n = 195) was 25.1 ± 5.7 years (range: 16–40 years) and the majority of them (163, 83.6%) were men. Potential female study candidates could often not be recruited for the study because they refused to undergo clinical examination. All enrolled study participants came from in total 18 sub-Saharan African countries (54.3% came from East Africa, 35.9% from West Africa, 10.8% from Central Africa – Table 1) and they were in Italy for a mean period of 54 days (range: 19–121 days).

**Table 1:** Summary of country of origin, number of cases included, 18S NASBA positive cases and gametocyte carriers

Country	Total number of cases included in the study	Total number of 18S NASBA positive cases from the respective country	Gametocyte carriers as assessed by Pfs25 NASBA
Ivory Coast – West Africa	14	3	1
Togo – West Africa	3		
Guinea – West Africa	8	2	
Somalia – East Africa	37	11	1
Ethiopia – East Africa	1		
Eritrea – East Africa	63	18	2
Ghana – West Africa	20	6	3
Nigeria – Central Africa	17	8	1
Burkina Faso – West Africa	7	1	1
Mali – West Africa	9	6	3
Cameroon – Central Africa	1		
Liberia – West Africa	3	2	1
Gambia – West Africa	4	1	
Chad – Central Africa	2		
Senegal – West Africa	2	1	
Sudan – East Africa	3	2	
Congo – Central Africa	1	1	

Total	195	62	13

Participants were allocated into three groups on basis of their reply to the questionnaire whether or not they had malaria in the past. Those people acknowledging one or more episodes of previous malaria in their country of origin were placed in group A: 58/195 (29.7%); subjects who said that they did not previously had malaria were placed in group B: 93/195 (47.7%); and individuals who could not remember a previous malaria infection were group C: 44/195 (22.6%).

EDTA-anticoagulated blood samples were labeled and temporarily stored for a maximum of 24 hours in a freezer bag before transportation to the laboratory, where the samples were stored a -80°C until processing for nucleic acids isolation. Thin and thick blood smears were prepared at the time of blood collection and stained with 10% Giemsa stain (pH 7.2).

### Molecular analysis of blood samples

#### Nucleic acid extraction

Blood samples (100 μl) were mixed with 900 μl of guanidium isothiocyanate lysis buffer before DNA and RNA isolation [26]. Thirty μl of silica dioxide was added to each sample after thorough mixing of the extraction mixture. Subsequently, nucleic acids bound to silica were washed twice with wash buffer (10 M GuSCN, 100 mM Tris-HCL pH6.4), twice with 70% ethanol, and once with acetone. Next, nucleic acids were eluted from the silica with 100 μl water and stored at -20°C.

#### 18S NASBA

Pan-*Plasmodium *specific NASBA targeting 18S rRNA [GenBank accession number M19172.1] of *Plasmodium falciparum*, *Plasmodium vivax*, *Plasmodium malariae *and *Plasmodium ovale *was performed on an IQ5 Real-Time analyzer (Bio-RAD) [23]. Amplification reactions were performed using Nuclisens Basic Kit (bioMèrieux) following the manufacturer's manual at a KCl concentration of 80 mM. The reaction mixture (5 μl) including primers and molecular beacon was incubated with the RNA extract (2.5 μl) at 65°C for two minutes and subsequently at 41°C for two minutes. 2.5 μl of Nuclisens enzyme mixture (AMV-RT, RNase H and T7 RNA polymerase) was added and amplification allowed for 90 minutes at 41°C.

The forward primer was: 5' TCAGATACCGTCGTAATCTTA 3' (nucleotides1066 to 1086); the reverse primer was: 5'-AATTCTAATACGACTCACTATAGGGAGAAGGAACTTTCTCGCTTGCGCGAA-3' (T7 promoter sequence, linker and nucleotides 1216 to 1235); the Pf18S molecular beacon was: 5'-FAM- CGATCG-GAGAAATCAAAGTCTTTGGG-CGATCG-DABSYL-3' (molecular beacon stem of 6 paired nucleotides and nucleotides 1182 to 1201). Time to positivity (TTP), i.e. the time point during amplification at which the number of target amplicons detected became higher than the mean for two negative controls plus 20 standard deviations, was calculated. Standard ring stage parasite dilution series were used as positive controls. A sample containing only water and reactions mixture was used as negative control. The analysis of samples and appropriate positive and negative controls were performed in duplicate.

#### Pfs25 NASBA

18S rRNA NASBA positive samples were also analysed for gametocyte specific Pfs25 mRNA QT-NASBA [25]. Briefly, real-time QT-NASBA for Pfs25 mRNA [GenBank accession number AF193769.1] was performed on a NucliSens EasyQ analyzer (bioMèrieux) using the Nuclisens BasicKit for amplification according to the manufacturer's manual at a KCl concentration of 80 mM and the same conditions of Real-Time 18S QT_NASBA.

The forward primer was 5'-GACTGTAAATAAACCATGTGGAGA-3' (nucleotides 204 to 227); the reverse primer was 5'-AATTCTAATACGA CTCACTATAGGGAGAAGG-CATTTACCGTTACCACAAGTTA-3' (T7 promoter sequence, linker and nucleotides 338–359); the PfS25 molecular beacon was 5'-Texas-Red-CGATCG-CCCGTTTCATACGCTTGTAA-CGATCG-DABSYL-3' (molecular beacon stem of six paired nucleotides and nucleotides 259–278). Time to positivity was calculated, i.e. the time point during amplification at which the fluorescence detecting target amplicons exceeded the mean fluorescence of two negative controls plus 20 standard deviations. Standard gametocytes dilution series were used as positive controls. The samples and the positive controls were performed in duplicate.

### Statistical analysis

In order to value the statistical association between history of malaria and signs and symptoms (i.e. splenomegaly, hepatomegaly, jaundice, urticaria, rales, leukopaenia, normocytic haemolytic anaemia, transaminase level) contingency double-entry tables were used and Odds Ratio and relative 95% Confidence Intervals and X^2 ^value were calculated. A p value of < 0.05 was considered statistically significant.

In order to evaluate the statistical association between 18S NASBA and the following variables: sex, regions of origin (West Africa, Central Africa, East Africa), previous episodes of clinical malaria as well as current clinical signs (i.e. splenomegaly, hepatomegaly, jaundice, urticaria, rales, leukopaenia, normocytic haemolytic anaemia, transaminase level) contingency double-entry tables were used and Odds Ratio and relative 95% Confidence Intervals and X^2 ^value were calculated. The t-Student's method was employed for the comparative evaluation of quantitative variables (continuous variables- i.e: age). A p value of < 0.05 was considered statistically significant. In order to value the potential confounding effect of considered variables (sex, age, regions of origins, history of malaria, current clinical symptoms and signs) on 18S NASBA positivity logistic regression model was performed.

## Results

### Clinical features

All examined study cases did not present at the time of examination or in the previous two months, the malaria paroxysm- characterized by high fever, chills and rigor. On physical examination 98/195 (50.2%) cases were found to have splenomegaly and tender hepatomegaly; 50/98 (51.0%) were in group A, 27/98 (27.6%) in group B and 21/98 (21.4%) in group C. Splenomegaly as well as hepatomegaly was significantly associated with group A (splenomegaly: OR 2.71, 95%CI = 1.36–5.48; χ^2 ^= 9.53, p < 0.05; hepatomegaly: OR = 3.01, 95%CI = 1.16–7.76; χ^2 ^= 6.8, p < 0.05). However, an inverse relationship was found between spenomegaly and group B (OR = 0.48, 95%CI = 0.26–0.89; χ^2 ^= 6.28, p < 0.05).

Other physical findings that have been observed are: jaundice 25/195 (12.8%), scleral icterus 61/195 (31.3%), urticaria 37/195 (19.9%). Auscultation of the chest revealed scattered rales in about 25% of subjects. Cardiac examination was generally normal except for tachycardia in about 10% of patients.

Some abnormalities in routine laboratory tests were found in some study participants. Normocytic haemolytic anaemia presented in 61/195 (31.3%) of the cases; leukopaenia due to a decrease in granulocytes and lymphocytes was present in 96/195 (49.2%) and these cases also presented with eosinophilia in the presence of urticaria. Leukopaenia was significant associated with group A (OR = 2.19, 95%CI = 1.11–4.36; χ^2 ^= 6.05, p < 0.05). Liver function tests revealed elevated transaminase levels in 32/195 (16.4%) of the cases and a mild to moderate increase in bilirubin (mostly indirect) in 86/195 (44.1%) of the study subjects; this variable resulted inversely associated with group C (OR 0.09, 95%CI = 0.002–50.56; χ^2 ^= 8.28, p < 0.05).

Serological markers for HIV were found positive in 0.8% of the enrolled cases, for HIV/HBV in 0.4% of the study individuals, for HIV/HCV in 0.6% of the cases, for HBV in 9.7% of the subjects and for HCV in 1.1% of the cases. Luetic infection was present in 6.4% of examined study subjects.

### NASBA

In total, 62 study participants (31.7%) were found positive for the presence of *Plasmodium *nucleic acids as revealed with the applied NASBA technique. These cases were distributed as follows: 18/58 (31.0%) belong to Group A, 28/93 (30.1%) belong to Group B and 16/44 (36.4%) belong to Group C. The distribution according to country of origin is presented in Table 1.

Microscopy confirmed the positive NASBA results in 14 cases and these cases were distributed as follows: six belonged to Group A, six belonged to Group B and two belonged to Group C. Quantification of parasitaemia was not available.

*Plasmodium falciparum *was observed in 13 samples and one sample was identified as being *P. ovale*. Thirteen cases (21%) of this *Plasmodium*18S rRNA positive population were also found positive with a Pfs25 mRNA NASBA, which is specific for the detection of gametocytes of *P. falciparum*. Four cases positive for gametocytes were confirmed by microscopy.

No significant association (p > 0.05) was found between 18S NASBA positivity and sex, age, history of malaria and region of origins.

A statistically significant association was found between 18S NASBA positivity and splenomegaly (χ^2 ^= 89.97, p < 0.001), hepatomegaly (χ^2 ^= 79.54, p < 0.001) and leukopenia (χ^2 ^= 89.97, p < 0.001): in all subjects who were found positive with the 18S NASBA, splenomegaly, hepatomegaly and leukopaenia were reported. The relative OR values were not computable. No other signs and symptoms was associated with 18S NASBA positivity.

By logistic regression model, a significant association was found between 18S NASBA and country of origin (p < 0.05). In particular, a high probability to be positive at 18S NASBA was observed in people coming from West Africa (OR: 33.8; 95%CI: 1.1–1085.5), Central Africa (OR: 56.7; 95%CI: 1.2–2598.9) and East Africa (OR: 235.6; 95%CI: 3.9–14290.9). By using the same regression model, a significant inverse association was found between increased transaminase levels and 18S NASBA positivity (OR: 0.14; 95%CI: 0.03–0.64; p < 0.05) so that people with increased transaminase levels had a lower probability to be positive at 18S NASBA. By the model, no significant associations (p < 0.05) were found between 18S NASBA positivity and sex, age, history of malaria, other signs and symptoms (splenomegaly, hepatomegaly, jaundice, urticaria, rales, leukopaenia, normocytic haemolytic anaemia).

## Discussion

In many countries, the increase in imported malaria cases from different countries is due to the increasing number of people traveling to and from endemic areas, without taking appropriate prophylaxis or immigration from low income countries [27,28]. In the case of returning non-immune travelers, malaria infection is often very obvious with severe disease and sometimes fatal outcome. In contrast, people from disease endemic countries may have acquired semi-immunity, resulting in less serious to none manifestations of disease, as was also shown in Spain [28], and recently also in Italy [29]. However, a proportion of this population may develop disease over time due to weaning immunity and/or contribute to possible transmission. In general, people originating from malaria endemic countries may have sub-microscopic levels of parasitaemia, but may feel not ill at all or present typical features of malaria. This is also reflected in the present study. Although many study participants could not recall having had malaria in the past, when being interviewed, there was no difference between the levels of infection between these participants and those who could recall an episode of malaria. In a substantial proportion of the examined population low levels of *Plasmodium *parasites were found with the applied NASBA technology. Furthermore, clinical examination revealed a statistically significant association between splenomegaly and 18S NASBA positivity. Malarial splenomegaly is believed to be a consequence of an immunological dysfunction due to recurrent episodes of malaria [30,31]. It is generally considered that prolonged exposure to malarial parasites (5–10 years) is needed to develop splenomegaly, this response is based on a overproduction of polyclonal IgM antibodies, leading to accumulation of high molecular-weight immunocomplexes and complement consumption. Long-term purification of immunocomplexes induces progressive hyperplasia of the spleen [32,33]. Patients usually present hypersplenism, and may have anaemia, leukopaenia, thrombopaenia and even haemolytic crises, depending on the severity [34]. A significant inverse association was found between increased transaminase levels and 18S NASBA positivity; i.e. people with increased transaminase levels had a lower probability to be positive at 18S NASBA. However, the high prevalence of patients with viral hepatitis infections observed in this study could also be the consequence of other diseases.

It was further noted in the present study that 21% of the *Plasmodium*18S rRNA positive population was also found positive with a Pfs25 mRNA NASBA, which indicates that these cases harbour gametocytes and may thus contribute to possible malaria transmission, even if the number of gametocytes is very low.

In Italy, malaria was eradicated after a campaign launched in 1947 just at the end of World War II. The campaign was carried out by indoor treatment with DDT; houses, stables, shelters and all rural structures were treated into the mid-1950s and also later in some endemic regions, such as Sardinia. Due to the persistence of sporadic malaria cases in some areas [35,36], the WHO declared Italy free of malaria only in 1970. In order to circumvent the potential reintroduction of malaria, a strict surveillance system was set up, which is still in place and governed by the Ministry of Health and the Istituto Superiore di Sanità (ISS, Rome, Italy), following WHO guidelines [37].

Over the last two decades, with the exception of one autochthonous case registered in 1997 in a rural area of the province of Grosseto (Tuscany), which was followed by four more cases (i.e. a cryptic case by *P. falciparum *in 2003 [38], two cases due to organ transplantation in 2004 [39] and a transfusional case in 2005, all cases of malaria have been imported [40-42]). Until to 1999 the number of imported cases of malaria arose constantly, while from 2002 to 2006 a decrease between 1% and 7% per year has been registered [37] in both immigrant (-9%) and among Italian citizens(-28%). Most of the cases registered from 2002–2006 were among people from Africa (88.4%), and, in particular, from Nigeria (26%), Ghana (17%), Senegal (17%), Costa d'Ivoire (12%), and Burkina Faso (6.7%). Furthermore, additional cases were reported in 2000 [43] and in 2002 [44] in Chinese immigrants who traveled to Italy via Africa. *Plasmodium falciparum *was confirmed to be the most prevalent species imported in southern Europe [27,28,37]; in Italy, this species is responsible for 89% of the infection, *P. vivax *for 7.7%, *P. ovale *and *P. malariae*, 6% and 1.5%, respectively [29,37]. In addition, mixed *Plasmodium *infections have also been reported in Italy, with 58% of all these cases reported from a single town, Verona [29].

The distribution and density of the three potential vectors of malaria in Italy (*Anopheles labranchiae, Anopheles superpictus *and *Anopheles sacharovi*) are also under strict surveillance by Istituto Superiore di Sanità (ISS). *Anopheles labranchiae *is present in several areas of Central and Southern Italy and in some regions (Tuscany, Calabria, Puglia, Sardinia and Sicily) high density have been registered [41]. The density of *An. superpictus *is lower and breeding sites are restricted to Calabria coasts and Sicily while although *An. sacharovi *has not registered since over 30 years, its presence in low density cannot be excluded in Apulia and northern Sardinia regions [37].

The combination of the presence of potential vectors and gametocyte carriers may pose a risk for re-introduction of malaria in the country. However, it should be noted that certain climatological and ecological prerequisites should probably be also met before the disease is really reintroduced [37].

## Conclusion

This study showed that a substantial proportion of people originating from malaria endemic countries and not showing clinical signs of malaria harbor *Plasmodium *parasites in their blood (i.e. 32% of positivity to *Plasmodium *spp., 21% of population harboring *P. falciparum *gametocytes). If transmission prerequisites (e.g. vector, susceptible population and climatological conditions) prevail they could potential be a reservoir. Therefore, health authorities should pay special attention to the health of this potential risk group and aim to improve their health conditions.

## Competing interests

The authors declare that they have no competing interests.

## Authors' contributions

MM was involved in the laboratory work (blood processing, microscopy and NASBA analysis testings). RDT was involved in the fieldwork (blood sample collection). DM was involved in statistical analysis. VF and GA were involved in clinical consultancy. PFM performed daily supervision, analysis and technical assistance of the NASBA assays and substantial revision of the manuscript. HDFHS, conception of the study, critical reading and revision of the manuscript. AG and GS, scientific and financial coordinators of the project and writers of the paper.
